# Recovery of activities of daily living in COVID-19 patients requiring intensive care unit or medical care unit: an observational study on the role of rehabilitation in the subacute phase

**DOI:** 10.3389/fresc.2023.1256999

**Published:** 2023-11-10

**Authors:** Chiara Notarstefano, Federica Bertolucci, Mario Miccoli, Federico Posteraro

**Affiliations:** ^1^ Department of Translational Research and New Technologies in Medicine and Surgery, DS Neurorehabilitation, University of Pisa, Pisa, Italy; ^2^ Department of Rehabilitation, Versilia Hospital, AUSL Toscana Nord Ovest, Lucca, Italy; ^3^ Department of Clinical and Experimental Medicine, University of Pisa, Pisa, Italy

**Keywords:** COVID-19, rehabilitation, physiotherapy, speech therapy, outcome, ICU, MCU

## Abstract

**Purpose:**

This study aims to describe the functional status of a cohort of subacute COVID-19 patients treated in a dedicated rehabilitation unit and to compare functional outcomes between patients previously hospitalized in the intensive care unit (ICU group) and patients assisted in the medical care unit (MCU group).

**Materials and methods:**

Clinical and functional evaluations were performed at admission and discharge. The functional status was assessed using Barthel index (BI), functional ambulation categories (FAC), trunk control test (TCT), and dysphagia outcome and severity score (DOSS). All patients received multidisciplinary tailored rehabilitation.

**Results:**

We evaluated 171 patients (with a mean age of 67.7 ± 11.9 years, 117 were males), 110 coming from the ICU (with a mean age of 63.24 ± 10.9 years), and 61 coming from the MCU (with a mean age of 75.75 ± 9.09 years). The ICU group showed a worse functional status at admission compared with the MCU group [BI 2.5 (0–20) vs. 20 (10–60), FAC 0 (0–0) vs. 0 (0–2), TCT 61 (42–100) vs. 100 (61–100), DOSS 5 (1–7) vs. 7 (7–7)] and had significantly longer hospital stay. At discharge, all functional scales were improved with no statistically significant differences between the two groups.

**Conclusion:**

Early rehabilitation of COVID-19 survivors improves functional recovery closing the initial gap between the ICU and MCU groups. In addition, it is effective to improve the functional outcome reducing the costs for longer-term assistance of COVID-19 patients.

## Introduction

COVID-19 is a systemic inflammatory disease primarily affecting the lungs with secondary involvement of multiple body systems, resulting in complex disability (1). The severity spectrum spans from mild respiratory syndrome accompanied by fever, dyspnea, and dry cough to a more severe end of acute respiratory distress syndrome (ARDS), which requires extended stays in the intensive care unit (ICU) (2).

Risk factors for mortality in COVID-19 include advanced age and comorbidities such as diabetes, obesity, systemic hypertension, renal impairment, coronary artery disease, and malignancies (3), but even individuals with lower susceptibility to COVID-19 mortality can develop disabling pulmonary and extra-pulmonary sequelae (2, 4). Disability arises from a combination of factors, including viral and inflammatory damage on multiple body structures, side effects of invasive supportive treatments, cognitive and psychiatric complications, and extended periods of immobilization. Neurological manifestations such as acute cerebrovascular disease, peripheral neuropathy, and myopathy can exacerbate impairments in motor function (5). Similar to other causes of critical illness, COVID-19 patients spending prolonged periods of immobility in the intensive care setting are at risk of developing ICU-acquired weakness (IAW) and post-intensive care syndrome (PICS). Both conditions can result in long-lasting physical and psychological sequelae, ultimately diminishing the overall quality of life (QoL) (6, 7). Nevertheless, since COVID-19 patients assisted in medical care units (MCU) can also display severe disability (8), the true impact of ICU-invasive supportive treatments on functional status has not been fully clarified yet. Furthermore, long-term sequelae of COVID-19, referred to as “long COVID,” encompass symptoms such as fatigue, muscle pain, palpitations, cognitive impairment, dyspnea, anxiety, chest pain, and arthralgia lasting for more than 12 weeks after the initial COVID-19 infection (9–11).

Starting from the early 2020, the COVID-19 pandemic has had an enormous impact on acute care facilities, with the implementation of ICU bed capacity and the transformation of general medical wards into specialized COVID units, referred to as COVID-dedicated MCU (12). Despite these changes, the reorganization of rehabilitation services has resulted in the creation of only a limited number of early subacute COVID rehabilitation units aimed to facilitate prompt functional recovery and to release pressure on acute care beds. Early rehabilitation for subacute COVID-19 patients is endorsed by various expert panels and clinical guidelines (13, 14), even though treatments are mainly addressed toward respiratory impairment rather than the broader range of functional deficits arising from the SARS-CoV-2 infection. Following the ventilatory failure phase, patients with COVID-19 pneumonia require a multidisciplinary rehabilitation approach focused on restoration of premorbid cardiopulmonary and motor function, thus allowing the recovery of independence in performing the activities of daily living (ADLs) (15). While interventions by speech and language therapist are essentials for post-ICU patients with prolonged orotracheal intubation and tracheostomy, milder forms of dysphagia have also been reported in non-invasively ventilated COVID-19 patients (16).

In the past few years, a limited number of observational studies have highlighted the positive role of early inpatient rehabilitation for post-COVID-19 disability (8, 17–20). These studies have provided insights into the baseline functional characteristics of COVID-19 patients and the outcomes of comprehensive rehabilitation treatments during the initial waves of the pandemic. As yet, among different studies, rehabilitation outcomes vary greatly due to relatively small samples of patients, heterogeneous rehabilitation protocols, and methodology of functional assessment. Recent systematic reviews, such as the one conducted by the Cochrane Rehabilitation Field (21), revealed that a greater body of research has been focusing on improving acute respiratory symptoms and understanding the long-term consequences of COVID-19 infection. Thus, the role of an early subacute comprehensive rehabilitation treatment and the comparison of global functional outcomes in patients previously requiring ICU care and those managed in MCU settings remains only partially elucidated.

In this article, we evaluated a large cohort of patients with COVID-19 pneumonia admitted to a dedicated subacute rehabilitation unit, with the aim of:

- Providing an accurate description of global functional outcomes focusing on the recovery of pulmonary function, swallowing, trunk control, walking abilities, and independence in activities of daily living at discharge.
- Comparing rehabilitation outcomes between patients previously hospitalized in intensive care units (ICU group) and patients assisted in medical care units (MCU group).

## Materials and methods

The case records of consecutive patients with COVID-19 pneumonia admitted to the rehabilitation unit of the Versilia Hospital between 1 September 2020 and 31 October 2021 were revised for this retrospective observational study.

The inclusion criteria were as follows:

• Age 18 years or older;
• Diagnosis of COVID-19 pneumonia confirmed by suggestive radiological changes on chest CT scan and current or previous laboratory confirmed SARS-CoV-2 infection;
• Severe respiratory failure treated in the acute phase with conventional or high flow oxygen, non-invasive ventilation (NIV), continuous positive airway pressure (CPAP), invasive mechanical ventilation, or extracorporeal membrane oxygenation (ECMO);
• Current hemodynamic stability without catecholamine infusion and respiratory stability with no ventilation, even if the patients needed the delivery of high oxygen flow with FiO2 of up to 60%;
• Sufficient autonomy in ADLs before hospitalization proved by anamnestic Barthel index (BI) > 50;
• Ongoing severe, complex motor/respiratory disability due to SARS-CoV-2 infection requiring intensive inpatient rehabilitation.

Patients eligible for rehabilitation inpatient stay for orthopedic or neurological conditions (such as fractures, major surgery, stroke, severe brain injury) with coincident asymptomatic SARS-CoV-2 infection were excluded. Patients with SARS-CoV-2-related neurological complications occurring during the acute phase of COVID-19 pneumonia were enrolled. This study was performed in accordance to the STROBE (Strengthening the Reporting of OBservational studies in Epidemiology) checklist.

The study was approved by the local ethical committee (Prot. No. 19164) as an observational study.

### Data collection

#### Anamnestic and clinical data

Demographic, anamnestic, and general clinical data were collected using an electronic worksheet. The presence of morbid obesity, diabetes, chronic lung disease, and chronic neurological disorders was selected as premorbid anamnestic data. The functional status of the patient before hospitalization was assessed using the anamnestic BI (22) and functional ambulation classification (FAC) (23). Anamnestic functional parameters were obtained by interview of relatives and caregivers. Cumulative illness rating scale (CIRS) was used to quantify the burden of comorbidities (24). COVID-19 vaccination status (single dose, booster dose, or not vaccinated) of the included patients was recorded. Clinical data included the intensity of care (ICU or MCU) during the acute hospitalization, acute care length of stay (LOS), rehabilitation LOS, bacterial superinfections, and presence of COVID-19-related neurological manifestations. The diagnosis of peripheral nervous system complications required EMG/ENG confirmation. All of the following data were assessed at the time of admission and discharge: pulmonary gas exchange as assessed by PaO_2_/FiO_2_ (P/F) calculated through arterial blood gas analysis, need for oxygen supplementation, dysphagia, and artificial nutrition support.

#### Rehabilitation outcomes and interventions

Discharge destination of the patient and referral to outpatient rehabilitation were recorded. Trunk control impairment was assessed by trunk control test (TCT) (25). This indirect measure of motor function was adopted as most patients were bedridden at admission to the rehabilitation unit. Severity of swallowing impairment was evaluated using the dysphagia outcome and severity scale (DOSS) (26). Patients were considered dysphagic with a DOSS score of ≤5 (5–3 indicates the need of dietary changes, ≤2 indicates the need for artificial nutrition). Walking abilities of the patient were assessed using the FAC, while Barthel index was used as a measure of independence in ADLs. All functional scales were administered at admission and at discharge from the rehabilitation unit. Improvement during rehabilitation was reported by changes of functional outcome measure scales from admission to discharge (ΔTCT, ΔDOSS, ΔFAC, ΔBI). Data on prescription of ambulatory assistive devices were collected as well. Physical performance measures such as the 6-min walking test (6MWT) (27) and the short physical performance battery (SPPB) (28) were available on a very limited number of clinical records and will not be presented.

All patients underwent daily, individual, or group sessions of multidisciplinary rehabilitation which included the following domains of interventions:

• Pulmonary rehabilitation: training for breath control and diaphragmatic re-education, chest expansion and volume increasing exercises, secretion clearance by bronchus suction and positive expiratory pressure (PEP), or cough assistive devices
• Motor rehabilitation: active-assisted and active joint mobilization of the four limbs, muscle strength training, exercises for trunk control, and recovery of postural reflexes; resumption of standing position and walking; reconditioning exercise program with interval training and continuous aerobic training, prescription of orthosis, and walking aids
• Swallowing rehabilitation: sensory–motor stimulation, postural compensation, modified consistency diet, and oral hygiene

An isolated dedicated space including fully equipped gym rooms for comprehensive rehabilitation treatment was arranged. A specific pool of physiotherapists specialized in motor and respiratory rehabilitation together with a language and speech therapist specialized in swallowing rehabilitation and assessment of cognitive dysfunctions was involved. All rehabilitation professionals were provided with complete personal protective equipment (including surgical and FFP2 masks, gloves, goggles, glasses, face shields, gowns, and aprons). Due to significant discomfort caused by personal protective equipment, rehabilitation therapists were able to stay inside the dedicated ward for no more than 3 h/day, thus resulting in increased length and costs of treatments and great distress for therapists.

Despite this, the overall duration of daily rehabilitative session was 3 h per patients divided into 2 h of individual treatment and 1 h of group sessions. The latter were reserved to patients with better performance status.

### Statistical analysis

Descriptive data were reported as frequencies, medians, means, interquartile ranges (IQRs), and standard deviations. Shapiro–Wilk test was used to verify the normality of the distributions. Student's *t*-test and Mann–Whitney *U* test were performed to compare quantitative and ordinal variables. Categorical variables were compared using the Chi-squared test or Fisher's exact test. *P*-values of <0.05 were considered statistically significant. Statistical analysis was carried out with R 4.0.3 for Windows and XLSTAT 2020.

## Results

### Demographic and premorbid anamnestic data

One-hundred and seventy-one patients with COVID-19 pneumonia were admitted to our rehabilitation unit between 1 September 20 and 20 October 21. Demographical and premorbid anamnestic data are represented in Table 1.

**Table 1:** Demographical and premorbid anamnestic data in the study population, ICU group, and MCU group.

	Study population (171)	ICU group (110)	MCU group (61)	*p*-value
Age (mean, SD)	67.7 ± 11.9	**63.24** ± **10.9**	**75.75** ±** 9.09**	**<0**.**0001**
Sex (M/F)	117/54	77/33	21/40	0,671
CIRS comorbidity (median, IQR)	2 (1–3)	**2** **(1–2.75)**	**2** **(1–3)**	**0**.**026**
CIRS severity (median, IQR)	1.38 (1.23–1.46)	**1.31** **(1.15–1.46)**	**1.41** **(1.30–1.54)**	**0**.**003**
Obesity (*n*, %)	45/171 (26%)	**35/110 (31.8%)**	**10/61 (16.4%)**	**0**.**044**
Diabetes (*n*, %)	44/171 (26%)	30/110 (27.3%)	14/61 (22.9%)	0.662
Respiratory disease (*n*, %)	30/171 (18%)	16/110 (14.5%)	14/61 (22.9%)	0–240
Neurological disease (*n*, %)	19/171 (11.1%)	**5/110 (4.5%)**	**14/61 (22.9%)**	**0**.**001**
FAC anamnestic (median, IQR)	5 (5–5)	**5 (5–5)**	**5 (4–5)**	**<0**.**0001**
BI anamnestic (median, IQR)	100 (100–100)	**100 (100–100)**	**100 (90–100)**	**0**.**0003**

CIRS, cumulative illness rating scale, FAC, functional ambulation classification, BI, Barthel index, IQR, interquartile range.

*p*-value refers to the comparison between the ICU group and the MCU group. Statistically significant differences are highlighted in bold.

The patients’ mean age at the time of hospitalization was 67.7 ± 11.9 years, and 117 of them were males. The median CIRS comorbidity was 2 (IQR 1–3) indicating two body apparatus/systems affected by the disease which requires therapy. CIRS severity, indicating the overall severity of comorbidities, was 1.38 (IQR 1.23−1.46). Obesity and diabetes had the same prevalence (26%) in the population. Approximately 18% of patients were affected by chronic respiratory disease, while 11.1% had a previous neurological disease. The median FAC anamnestic score was 5 (IQR 5–5), and the median anamnestic BI was 100 (IQR 100–100). Prior to hospitalization, only eight patients had received a single dose of COVID-19 vaccine, while none of the subjects included had a booster dose. The majority of patients (110 out of 171, 64.3%) were previously admitted to the ICU (ICU group), while 61 patients belonged to a COVID-19 medical unit (MCU group). The mean age was significantly higher in the MCU group compared with the ICU group (75.75 ±  9.09 vs. 63.24 ±  10.9, *p* < 0.0001). In the MCU group, higher CIRS comorbidity and severity scores were found, paired with significantly higher frequency of chronic neurological diseases. In the ICU group, a larger proportion of patients were morbidly obese, whereas no difference was observed in terms of the frequency of diabetes and chronic respiratory diseases. The premorbid functional state was significantly different in the two groups with higher anamnestic BI and FAC scores reported in the ICU group.

### Patient's clinical features

Significant clinical features prior to rehabilitation admission and duration of hospitalization in acute care and rehabilitation settings are reported in Table 2.

**Table 2:** Clinical features prior to rehabilitation admission in the study population, ICU group, and MCU group.

	Study population (171)	ICU group (110)	MCU group (61)	*p*-value
Acute care LOS, days (mean, SD)	35.5 ± 21.6	**40.63 **±** 23.22**	**26.25 **± **14.03**	**<0.0001**
Rehabilitation LOS, days (mean, SD)	19.9 ± 12.5	**22.1 **±** 13.3**	**16.13 **± **9.5**	**0.001**
Bacterial superinfections	115/171 (67.2%)	**88/110 (80%)**	**27/61 (44.2%)**	**0.0001**
NIV (*n*, %)	39 (22.8%)	**16 (14.5%)**	**23 (37.7%)**	**0.0007**
Orotracheal intubation (*n*, %)	—	92 (83.3%)	—	—
ECMO (*n*)	—	2	—	—
Tracheostomy (*n*, %)	—	83 (75%)	—	—

LOS, Length of stay; NIV, non invasive ventilation; ECMO, extracorporeal membrane oxygenation; SD, standard deviation.

*p*-value refers to the comparison between the ICU group and the MCU group. Statistically significant differences are highlighted in bold.

The mean LOS was 35.5 ± 21.6 days in acute care and 19.9 ± 12.5 days in rehabilitation. In the ICU group, both acute care LOS and rehabilitation LOS were significantly longer compared with the MCU group. Two significant outliers were found from the ICU group, who were hospitalized for 138 and 148 days due to difficult respiratory weaning and multiorgan failure. Hospital-acquired infections requiring antimicrobial therapy occurred more frequently in the ICU group.

Relevant clinical features at the time of admission and discharge from the rehabilitation unit are summarized in Table 3.

**Table 3:** Clinical features at the time of admission and discharge from the rehabilitation unit in the study population, ICU group, and MCU group.

	Study population (171)	ICU group (110)	MCU group (61)	*p*-value
Oxygen support, admission (*n*, %)	142/171 (83.4%)	96/110 (87.3%)	46/61 (75.4%)	0.077
Oxygen support, discharge (*n*, %)	66/171 (38.6%)	42/110 (38.2%)	24/110 (39.3%)	0,989
P/F, admission (mean, SD)	285.1 ± 106.2	283.05 ± 105.62	289.02 ± 106.4	0.551
P/F, discharge (mean, SD)	356.8 ± 95.2	357.2 ± 84.2	356.05 ± 114.9	0.941
Tracheostomy, admission (*n*, %)	—	68 (61.8%)	—	—
Tracheostomy, discharge (*n*, %)	—	11 (10%)	—	—
Dysphagia (DOSS ≤5), admission (*n*, %)	74/171 (43.3%)	**68/110 (61.8%)**	**6/61 (9.84%)**	**<0**.**0001**
Dysphagia (DOSS ≤5), discharge (*n*, %)	18/171 (10.5%)	14/110 (12.8%)	4/61 (6.6%)	0.309

P/F, Pa02/FIO2; DOSS, dysphagia outcome severity scale; SD, standard deviation.

*p*-value refers to the comparison between the ICU group and the MCU group. Statistically significant differences are highlighted in bold.

When comparing the ICU and the MCU groups, no significant differences of respiratory functional status (oxygen support requirements and P/F ratio) were found both at the time of admission and discharge. In the MCU group, 23 (37.7%) patients required NIV, while the remainder received conventional or high flow oxygen therapy. In the ICU subgroup, 16 (14.5%) patients were treated with NIV, and two patients were placed on ECMO, while 92 (83.3%) patients required orotracheal intubation with a mean length of endotracheal tube ventilation of 10.7 ± 4.6 days. A total of 83 (75%) patients had respiratory weaning with tracheostomy with a mean length of tracheostomy tube placement of 37.6 ± 20.3 days. A total of 15 patients were weaned from the tracheostomy tube before rehabilitation admission. Successful decannulation during rehabilitation occurred in 57 patients, whereas 11 patients were discharged with tracheostomy tube. Recovery of spontaneous breathing of mechanically ventilated patients was achieved at 11.22 ± 13.7 days before transfer to the rehabilitation unit. Only one patient had failed the invasive ventilation weaning and was discharge from the ICU with tracheostomy and long-term home mechanical ventilation. Clinically significant dysphagia was more common in the ICU group. In our cohort, the frequency of COVID-19-related neurological complication was 6.4%, and no difference of frequency of central or peripheral nervous system manifestations was observed among the two groups. In the MCU group, there were two cases of COVID-19-related subacute polyneuropathy and one case of ischemic stroke occurring during the acute phase of COVID-19 pneumonia. Four patients from the ICU group were diagnosed with critical illness myopathy and neuropathy (CRIMYNE), and one patient presented with COVID-19-related subacute polyneuropathy. Two cases of ischemic stroke and one case of spontaneous intracranial bleeding occurred in the ICU group.

### Rehabilitation outcomes

Discharge destination and walking abilities are shown in Table 4.

**Table 4:** Discharge destination and walking abilities in the study population, ICU group, and MCU group.

	Study population (171)	ICU group (110)	MCU group (61)	*p*-value
Discharge destination				
Home (*n*, %)	145/171 (84.8%)	93/110 (84.5%)	52/61 (85.24%)	0.920
Outpatient rehabilitation (*n*, %)	31/171 (18.1%)	**27/110** **(24.5%)**	**4/61** **(6.5%)**	**0**.**007**
Inpatient rehabilitation (*n*, %)	11/171 (6.4%)	9/110 (8.2%)	2/61 (3.3%)	0.542
Acute care (*n*, %)	14/171 (8.2%)	7/110 (6.4%)	7/61 (11.5%)	0.257
Walking abilities				
Free walking (*n*, %)	65/171 (38.01%)	39/110 (35.5%)	26/61 (42.6%)	0.447
Walking aid (*n*, %)	84/171 (49.1%)	54/110 (49.1%)	30/61 (49.1%)	0.955
No functional walking (*n*, %)	22/171 (12.9%)	16/11 (14.5%)	6/61 (9.8%)	0.520

Statistically significant differences are highlighted in bold.

Clinical instability requiring transfer back to acute care occurred in 14 out of 171 patients (8.2%). No deaths were reported in the study population. Overall, 145 patients were discharged home, while 11 patients with partial functional recovery required further inpatient treatment in a non-COVID-19 rehab unit. Out of the 145 patients discharged home, 31 were referred to our outpatient rehabilitation facilities. No difference was found between the two groups in terms of the rate of discharge to home, transfer to lower-intensity inpatient rehabilitation, or transfer to acute care. A higher proportion of patients from the ICU group were referred for outpatient rehabilitation therapy. Approximately 88% of the study population recovered functional walking abilities with 65 patients who were able to walk with no ambulatory assistive device. Walking aid prescription and training were performed in 84 patients, while 12% of the patients were non-functional ambulators at the time of discharge. No differences were observed in the walking abilities at discharge between the two groups.

Rehabilitation outcome measure scales are presented in Table 5.

**Table 5:** Pre- and post-rehabilitation functional outcome measures.

	Study population (171)	ICU group (110)	MCU group (61)	*p*-value
TCT (admission)	84 (48–100)	**61** **(42–100)**	**100** **(61–100)**	**0**.**001**
TCT (discharge)	100 (100–100)	100 (100–100)	100 (100–100)	0.456
DOSS (admission)	7 (1–7)	**5** **(1–7)**	**7** (**7–7)**	**<0**.**0001**
DOSS (discharge)	7 (7–7)	7 (7–7)	7 (7–7)	0.204
FAC (admission)	0 (0–0)	**0** **(0–0)**	**0** **(0–2)**	**0**.**001**
FAC (discharge)	3 (2–4)	3 (2–4)	4 (2–4)	0.152
BI (admission)	10 (0–32.5)	**2.5** **(0–20)**	**20** **(10–60)**	**<0**.**0001**
BI (discharge)	70 (55–90)	70 (55–90)	70 (60–90)	0.391
ΔTCT	13 (0–39)	**24** **(0–52)**	**0** **(0–26)**	**0**.**001**
ΔDOSS	0 (0–4)	**2** **(0–6)**	**0** **(0–0)**	**<0**.**0001**
ΔFAC	3 (1–4)	**3** **(2–4)**	**2** **(1–3)**	**0**.**035**
ΔBI	50 (26.5–68.75)	**55** **(35–70)**	**40 (15–55)**	**0**.**001**

TCT, trunk control test; DOSS, dysphagia outcome severity scale; FAC, functional ambulation classification, BI, Barthel index.

All data are expressed as median, IQR. *p*-value refers to the comparison between the ICU group and the MCU group. Statistically significant differences are highlighted in bold.

**Figure 1:**
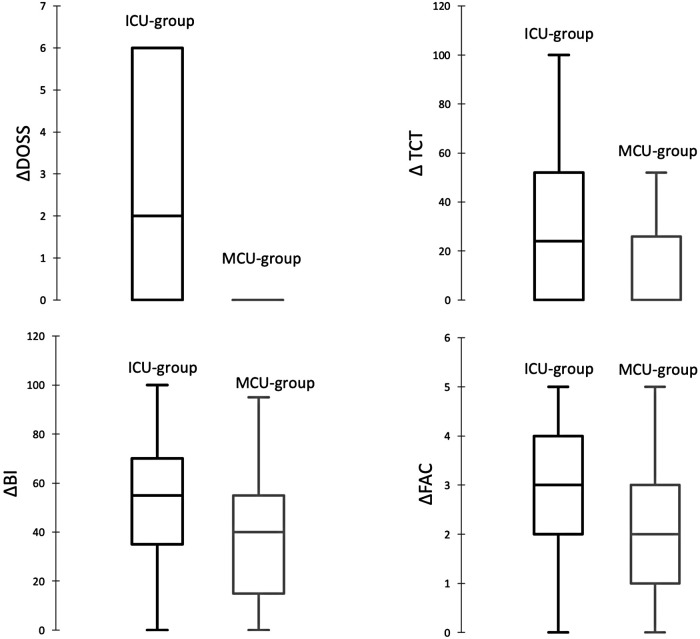
Box plots representing ΔDOSS, ΔTCT, ΔFAC, and ΔBI in the ICU and MCU groups.

In the study population, an improving trend was reported in motor impairment and dysphagia with complete recovery of trunk control (median admission TCT 100, IQR 100–100) and swallowing abilities (median admission DOSS 7, IQR 7–7). The median FAC at admission was 0 (IQR 0–0), which meant that on arrival to the rehab unit most patients were unable to walk. Following rehabilitation, two-thirds of the patients were able to walk with minimal physical assistance, supervision, or independently (median discharge FAC 3, IQR 2–4). Similarly, performance in ADLs improved from almost complete dependence at the time of admission (median admission BI 10, IQR 0–32.5) to minimally dependent levels at discharge (median discharge BI 70, IQR 55–90). Compared with the MCU group, at the time of admission TCT, DOSS, FAC, and BI were significantly lower in the ICU group. At the time of discharge, no difference was found in motor and swallowing impairment, functional dependence, and walking abilities between the two groups. Changes of functional measure scales from admission to discharge (ΔTCT, ΔDOSS, ΔFAC, ΔBI) were significantly more evident in the ICU group compared with those in the MCU group (Figure 1).

**Figure 2:**
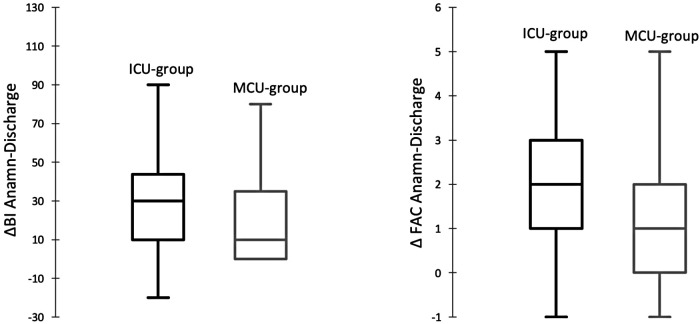
Box plot gap between anamnestic BI and BI at discharge (ΔBI Anamn-Discharge) and anamnestic FAC and FAC at discharge (ΔFAC Anamn-Discharge) in the ICU group and the MCU group.

In comparison to the MCU group, a greater difference between anamnestic BI and BI at discharge was reported in the ICU group (*p*-value = 0.004). Similarly, the discrepancy between anamnestic FAC and FAC at discharge was higher in the ICU group compared with that in the MCU group (*p*-value = 0.012) (Figure 2).

## Discussion

Since the early COVID-19 pandemic, a considerable amount of research has been published on early respiratory rehabilitation and long-term sequelae of SARS-CoV-2 infection (21). However, more limited evidence has been provided regarding the overall subacute functional limitations. In this paper, we provide an accurate description of baseline features and functional outcomes of a large cohort of hospitalized COVID-19 pneumonia survivors, undergoing a comprehensive rehabilitation program in a dedicated rehabilitation unit.

Following the acute phase, COVID-19 survivors display a complex disability with motor and gait impairment, swallowing difficulties, and loss of independence.

Upon arrival to our rehabilitation unit, patients presented severe motor skills deficiency with reduced trunk control (median TCT 84, IQR 48–100), inability to walk (median FAC 0, IQR 0–0), and inability to perform basic ADLs (median BI 10, IQR 0–30; mean, SD 20.5 ± 25.1). Dysphagia requiring logopedic interventions (DOSS < 5) affected approximately 43% of our study population, with median DOSS of 7 (IQR 1–7). Compared with similar datasets available in the literature, our study population presented with a higher degree of disability upon admission to the rehabilitation unit. A large retrospective multicentric study (29), collecting data on respiratory rehabilitation units across different hospitals in northern Italy, reported greater BI scores on admission (mean, SD 56.81 ± 31.49). Likewise, in a retrospective study carried out on 100 post-ICU COVID patients, Piquet et al. observed a mean BI of 77.3 ± 26.7 upon admission (17). Discrepancies in baseline disability levels might be attributed to more inclusive admission criteria of our rehabilitation unit, which enabled the early initiation of rehabilitation therapy for severely compromised patients with prolonged tracheostomy weaning and high oxygen requirements. In fact, our rehabilitation unit was able to admit subacute critically ill patients directly from intensive care departments across several regional hospitals, thus avoiding stepdown to low care medical wards and saving thousands of days of acute bed hospitalization. As a further confirmation of the markedly compromised clinical conditions, we found a significantly extended acute care LOS of over 30 days (mean, SD 35.5 ± 21.6) that could explain a more severe immobilization syndrome.

Despite the serious medical conditions, only 8.2% of the patients became clinically unstable and required transfer back to acute care. Furthermore, the sufficiently preserved premorbid functional status, with an anamnestic BI of >50 adopted as admission criteria, and the relatively moderate comorbidity burden (median CIRS comorbidity 2, IQR 1–3; median CIRS severity 1.38, IQR 1.23–1.46) are unlikely to justify the low performance status on admission.

According to our previous findings (20), this study confirms the benefits of an early multidisciplinary approach in recovering independence levels, thus allowing discharge to home with reduced caregiver burden. Specifically, discharge to home was achieved in 84.8% of the patients with 31 patients who continued rehabilitation treatments in an outpatient setting. Only 15% of the patients were transferred to a lower-intensity non-COVID rehab unit. Our discharge destination figures are quite similar to previous literature reports (17, 29, 30). Moreover, in our cohort, the rehabilitation treatment was associated with important motor function improvement with complete restoration of trunk control and consistent recovery of ambulatory capacities. The median FAC on discharge was 3 (IQR 2–4) meaning that 75% of the patients were able to walk with minimal physical assistance, simple supervision, or independently. Despite the relevant improvement, FAC at discharge was lower than premorbid levels (median anamnestic FAC 5, IQR 5–5). Walking aid training was required in 84 patients, while only 12% of the patients were non-functional ambulators at the time of discharge (FAC = 0). Swallowing abilities were restored in the majority of the cohort (median DOSS 7, IQR 7–7) with successful weaning from artificial nutrition in all patients and logopedic follow-up arranged for 19 subjects. After rehabilitation, functional independence in ADLs measured with BI improved up to a median score of 70 (IQR 55–90) although it did not reach pre-admission levels (median anamnestic BI 100, IQR 100–100). These findings are consistent with previous research reports (17, 20), highlighting the fact that despite the demonstrated efficacy of early subacute rehabilitation, a significant proportion of COVID-19 patients will still present some degree of functional impairment at discharge from hospital.

In order to evaluate the contribution of previous ICU-invasive treatment on subacute disability, we compared clinical features and rehabilitation outcomes in a subgroup of 61 patients arriving from COVID-19-dedicated medical units (MCU group) and 110 ICU survivors (ICU group).

Compared with the ICU group, MCU patients were significantly older (75.75 ± 9.09 vs. 63.24 ± 10.9, *p*-value < 0.0001) and presented a higher prevalence of comorbidities and prior limitations of ADLs. Younger-aged individuals admitted to the ICU during the COVID-19 pandemic were found in previous studies (8, 30, 31), and it is likely to represent a confounding factor. In fact, the huge demand of ICU beds during the pandemic waves may possibly have actually facilitated ICU admissions of younger patients with better performance status and favorable prognosis. As far as comorbidities are concerned, in our cohort, ICU and MCU patients displayed a similar prevalence of diabetes and chronic respiratory disease. By contrast, morbid obesity was more prevalent in the ICU group, and this could confirm obesity as a risk factor of severe and critical COVID-19 pneumonia requiring intensive care treatment (32).

More importantly, length of hospitalization, rate of hospital-acquired infections, and extra-pulmonary complications such as dysphagia were significantly more represented in the ICU group. Contrary to expectations, in our cohort, no significant differences between the ICU and the MCU group were observed with regard to COVID-19-related neurological complications. The prevalence of COVID-19-related neurological disorders is still uncertain in available literature (5) and was detected in just 6.4% of our study population. Given that neurophysiological and neuroimaging investigations were not easily available in COVID setting, it is possible that our data underestimated the real impact of neurological complications. As spirometry was contraindicated due to infection control reasons, respiratory functional status was investigated with oxygen support requirements and P/F ratio. Both parameters were not different between the ICU and MCU groups at admission and at discharge from the rehabilitation unit. Respiratory rehabilitation studies have reported that in the subacute phase oxygenation is only mildly compromised, whereas lung volumes and diffusion capacity studies are more sensitive in detecting COVID-19 respiratory dysfunctions (18, 29).

Compared with the MCU group, all functional parameters at admission (TCT, DOSS, FAC, and BI) were significantly reduced in the ICU group. By contrast, upon discharge, no difference was found in motor and swallowing impairment, functional dependence, and walking abilities between the two groups. In addition, in the ICU group, changes of functional measures from admission to discharge (ΔTCT, ΔDOSS, ΔFAC, ΔBI) were higher, thus indicating that despite worse baseline functional impairment ICU patients positively responded to the rehabilitation treatment. Our results are only partially in agreement with previous studies comparing ICU and MCU functional parameters. Two studies (31, 33) reported a worst functional outcome in terms of independence with ADL in COVID-19 patients hospitalized in the ICU, compared with subjects who did not require ICU treatment. In both studies, no inpatient subacute comprehensive rehabilitative treatment was provided. In the study by Leite et al., patients were treated by telemonitoring or received a physical exercise guide in a booklet and/or video format, while less than 8% of the patients were referred for individual physical therapist session. On the other hand, Piquet et al. (17) found comparable LOS and no significant differences of pre- and post-rehabilitation BI, sit-to-stand test, and handgrip strength between ICU and MCU patients. However, ICU patients improved more in terms of handgrip strength at discharge. Another retrospective study (8) reported prolonged LOS in ICU patients and similar functional state on discharge. It is increasingly recognized that, especially in ICU patients, COVID-19 disability is the result of the combination of respiratory dysfunction and motor impairment (19). To the best of our knowledge, this study is the first to report rehabilitation outcomes in such a large cohort of post-ICU COVID-19 patients. Our findings corroborate the hypothesis that functional impairment of COVID-19 is not only respiratory but is also strictly dependent on the severity of immobilization syndrome, extra-pulmonary complications, and side effects of invasive supportive treatments which could be amended by a rehabilitative treatment provided at an early stage. In our study, no difference was found between the two groups in terms of the rate of discharge to home, transfer to lower-intensity inpatient rehabilitation or acute care. A higher proportion of patients from the ICU group were referred for outpatient rehabilitation therapy. The latter evidence is consistent with the fact that ICU patients display a more pronounced gap between anamnestic and discharge functional measures. Therefore, early inpatient rehabilitation is associated with relevant improvement of swallowing, motor disability, and independence. Nonetheless, considerable disability persists at discharge, especially in ICU patients, suggesting the need for long-term treatment and follow-up.

Our study presents some limitations. First of all, the retrospective nature exposes to the risk of missing data and bias. Missing data on clinical records during the COVID-19 pandemic is a common consequence of increased workload for healthcare professionals and continuous patient turnover. Second, the lack of a control group of COVID-19 patients not receiving post-acute rehabilitation treatment does not allow to draw secure conclusions on specific effects of tailored multidisciplinary rehabilitation. Our study did not include spirometry evaluation or physical performance status tests, as the vast majority of patients were unable to perform a 6MWT of or SPPB either at admission or at discharge. Finally, our multidisciplinary assessment was lacking of formal neuropsychological screenings and QoL assessment that are of utmost importance for an extensive evaluation of post-intensive care sequelae.

In conclusion, the COVID-19 pandemic has posed new challenges for rehabilitation medicine in terms of support of subacute recovery and management of possible long-lasting effects on multiple body structures and functions.

Our study suggests that an early subacute rehabilitation of severely compromised COVID-19 patients is not only feasible but also crucial to improve clinical outcomes and independence in survivors, and it is also useful to release pressure on acute care beds. Post-ICU patients have more severe disability secondary to extra-respiratory and iatrogenic complications but can rely on chances of functional recovery that are similar to patients hospitalized in the MCU. This study confirms that the organization of comprehensive rehabilitation settings able to assist subacute patients, still positive for SARS-CoV-2 infection, represents an efficient healthcare systems answer to the catastrophic pandemic, decompressing acute hospital and improving the short-term outcome of severely impaired COVID-19 patients.

Even if the cost for the organization of this kind of rehabilitation units is higher (34), it could result in lower expenses for long-term assistance of post-COVID-19 patients.

In spite of early subacute rehabilitation, a significant proportion of patients still present some degree of functional impairment after discharge from hospital. Better characterization of prognostic factors of persistent functional impairment is required to identify patients at risk of incomplete recovery. Furthermore, future research should address changes of functional status from discharge to longer-term follow-up in order to address subsequent healthcare and rehabilitation needs of COVID-19 survivors and to assess the efficacy of early rehabilitation in reducing long COVID disability.

## Data Availability

The raw data supporting the conclusions of this article will be made available by the authors, without undue reservation.
